# Barriers and facilitators to the implementation of prehabilitation for elderly frail patients prior to elective surgery: a qualitative study with healthcare professionals

**DOI:** 10.1186/s12913-024-10993-2

**Published:** 2024-04-26

**Authors:** Tamina Isabel Fuchs, Carina Pfab, Jörn Kiselev, Stefan J Schaller, Claudia Spies, Tanja Rombey

**Affiliations:** 1 Berlin School of Public Health, Charité - Universitätsmedizin Berlin, Charitéplatz 1, 10117 Berlin, Germany; 2 Federal Institute for Occupational Safety and Health (BAuA), Nöldnerstraße 40-42, 10317 Berlin, Germany; 3 Department for Anesthesiology and Intensive Care Medicine (CCM/CVK), Charité - Universitätsmedizin Berlin, Corporate Member of Freie Universität Berlin, Humboldt-Universität zu Berlin, Berlin Institute of Health, Charitéplatz 1, 10117 Berlin, Germany; 4 Department for Health Sciences, Hochschule Fulda University of Applied Sciences, Fulda, Germany; 5 Department of Anesthesiology and Intensive Care Medicine, Klinikum rechts der Isar, School of Medicine and Health, Technical University of Munich, Ismaninger Str.22, 81675 München, Germany; 6 Department of Health Care Management, Technische Universität Berlin, Straße des 17. Juni 135, 10623 Berlin, Germany

**Keywords:** Prehabilitation, Frailty, Implementation, Barriers, Facilitators, Qualitative study

## Abstract

**Background:**

Prehabilitation aims to enhance functional capacity before surgery, minimise complications and achieve a better postoperative outcome. This can be particularly useful for older, frail patients to better tolerate surgery. The aim of this study was to identify what barriers and facilitators healthcare professionals in Germany experienced in the implementation and delivery of the multimodal prehabilitation programme “PRAEP-GO” for (pre-)frail adults aged 70 years and older to inform the implementation of prehabilitation into standard care.

**Methods:**

A nested descriptive qualitative study was conducted using semi-structured face-to-face interviews with healthcare professionals involved in the PRAEP-GO trial from the Berlin and Brandenburg region in Germany. Transcripts were analysed using Kuckartz’ qualitative content analysis. Results were interpreted and synthesised using the Consolidated Framework for Implementation Research, a theoretical framework to allow their application to a more general context.

**Results:**

A total of 14 interviews were conducted. Seven therapists (physio-, ergo-, sports therapy), five physicians and two employees from other professions with mainly administrative and organisational tasks in the project. All identified barriers and facilitating factors could be assigned to the themes of organisation, prehabilitation, cooperation and communication between healthcare professionals and with patients. Much optimisation potential was found regarding organisational aspects, e.g. addressing perceived staff shortages and optimising the patient pathway. Furthermore, it became apparent that communication and cooperation between professionals but also with patients need to be improved. More evidence regarding prehabilitation should be provided to convince professionals more. Prehabilitation should be multimodal and individualised, including the programme duration. Officially introducing prehabilitation into standard care would facilitate its delivery.

**Discussion:**

These findings underscore the fact that successful implementation of prehabilitation programmes, such as PRAEP-GO, requires sufficient organisational infrastructure, human resources, access to knowledge, an adaptable and individualised programme design as well as good communication among professionals and with patients. The transferability of the findings is limited by the absence of nutritionists and resulting overrepresentation of other therapists in the sample. To further convince professionals and patients of the concept of prehabilitation, more research is needed to build a solid evidence base that will ensure greater awareness and, thus, more motivation and cooperation among professionals and patients.

**Trial registration:**

Open Science Framework (osf.io/ksfgj).

**Supplementary Information:**

The online version contains supplementary material available at 10.1186/s12913-024-10993-2.

## Introduction

Due to the increasing life expectancy and ageing of society, the number of physically impaired patients with multiple comorbidities is rising and with it the proportion of older patients undergoing surgery [1]. In Germany, one-third of inpatient surgeries in 2020 were performed on people aged 70 and older. Of all age groups, most surgeries were performed in patients aged 75–80 years, with over 1,5 million surgeries [2].

A surgery generally represents a stress factor for patients that is associated with temporary deconditioning. Tolerance to surgery may be impaired, particularly in older patients, through age-related psychophysiological changes and comorbidities, increasing the risk of complications and negative health consequences for this patient group [3]. Especially frail older patients are more vulnerable for perioperative complications [4], postoperative complications, prolonged hospital stays, disability, and death [5]. Frailty can be described as a biological syndrome in which reserves and resistance to stressors decline, leading to a cumulative deterioration of multiple physiological systems [6–11]. It is an age-related condition of high vulnerability for adverse health outcomes like falls, disability, and delirium, in older adults [12–15].

Prehabilitation aims to help patients return to the highest possible level of function as quickly as possible after surgery [3]. It is a process of improving an individual’s functional capacity to withstand the stressors of inactivity [16], e.g. following surgery. Prehabilitation involves targeted preventive interventions like exercise training or nutrition therapy to improve health and health-related functions before an operation [3, 17], thereby speeding up recovery [18, 19], and reducing the length of stay in hospital [20] as well as postoperative complications [21]. In multimodal prehabilitation programmes, different interventions are combined [18], making it a complex and multidisciplinary intervention.

For successful implementation of a complex intervention such as multimodal prehabilitation, it is essential to find out which factors promote or hinder different components of the intervention. Apart from the participating patients, the view of the healthcare professionals involved in the prehabilitation process is particularly important because they are both experts for their workplace as well as prehabilitation itself. Furthermore, in multimodal prehabilitation, different professional groups across different areas must work together as a team that is characterised by the mix of different skills of its team members and potential skill-mix change [22, 23].

Previous studies identified several barriers to the implementation of prehabilitation from the perspective of healthcare professionals, such as a lack of human resources, lack of time to take an active role in prehabilitation as a physician, unclear financing, lack of communication between healthcare providers [24], the time for prehabilitation before surgery being too short [24, 25] or that exercise information were not well read or understood by patients [25]. However, these studies also found facilitators, including when there was an opportunity for healthcare professionals to talk about a healthy lifestyle with their patients [24], when prehabilitation had already been incorporated into standard care [24, 25], and when programmes allowed the exercises to be adjusted to the patient’s physical abilities and personal preferences and integrated in patient’s daily living [25].

The aim of this qualitative study was to identify, through qualitative expert interviews, what barriers and facilitators, specifically to the implementation and delivery of prehabilitation for frail elderly patients, healthcare professionals experienced in the context of the PRAEP-GO trial to inform the implementation of prehabilitation into standard care.

The PRAEP-GO multicentre randomised trial (NCT04418271, registered on 5 June 2020) currently investigates the (cost-)effectiveness of a multimodal prehabilitation programme for elderly (pre-)frail patients prior to elective surgery in Germany [26]. The trial enrolled approximately 1,400 patients across different regions over 3 years, and follow-up will end in August 2024 [27]. The overall goal was to inform the nationwide implementation of the PRAEP-GO prehabilitation programme into standard care in Germany.

## Methods

### Design

This was a nested descriptive qualitative study using semi-structured face-to-face interviews with health professionals involved in the ongoing multicentre randomised PRAEP-GO trial that investigates the (cost-)effectiveness of a multimodal prehabilitation programme for frail or pre-frail older people prior to elective surgery [26].

In the PRAEP-GO trial, patients were identified at participating hospitals during their initial consultation for an upcoming elective surgery with an expected duration of anaesthesia ≥ 60 min and without restriction on the treatment area. Interested patients over the age of 70 were screened for frailty using criteria by Fried et al. 2001 [12], and included in the trial if pre-frail or frail. When assigned to the intervention group, a three-week prehabilitation programme took place prior to surgery. Prehabilitation was planned individually for each patient during a shared decision-making (SDM) conference involving different healthcare professionals as well as the patient or their relatives. The trial was funded by the Innovation Fund of Germany’s Federal Joint Committee [28], meaning that the prehabilitation programme might be recommended for nationwide implementation should it prove to be (cost)effective [29].

The qualitative study presented here was performed in accordance with the Declaration of Helsinki and was approved by the ethics committee of the Charité – Universitätsmedizin Berlin (EA1/266/20, version 1.4) as well as the staff council. A protocol was prospectively registered on May 18, 2022 on the Open Science Framework (OSF) (osf.io/ksfgj). Reporting was guided by the Consolidated criteria for Reporting Qualitative research (COREQ) checklist [30] (appendix A).

### Study population

Inclusion criteria for interview participants were healthcare professionals of any age and gender who were involved in the PRAEP-GO trial and represented a mix of professions such as therapists, physicians and staff who mainly take on organisational tasks in the project, such as coordinating patients. Professionals who only carried out study-related tasks, such as outcome assessment, were excluded because we aimed to collect information about the prehabilitation process only. In that regard, purely study-related aspects, such as tests or documentation, were not of interest, as they will be omitted if prehabilitation is introduced into standard care. The interviewees were selected in the form of a convenience sample. For feasibility reasons, only healthcare professionals from the Berlin/Brandenburg region, where the trial lead is based, were included. There was no financial compensation for the interview participants.

Potential interview partners were reached via e-mail invitation. The mail addresses were obtained with the help of the official websites of the respective institutions. Members of the PRAEP-GO administration team supported the contacting process by passing on official e-mail distribution lists or contact information and disseminating information about the study. A total of 29 invitations for interviews were sent to reach all participating institutions of the PRAEP-GO project in Berlin/Brandenburg region to recruit professionals for the interviews. Interview partners were transparently informed about the study and data protection regulations, and their informed consent was obtained before the interview.

### Data collection

Data collection was conducted in the form of one-on-one interviews by one researcher, a female postgraduate student of Public Health with an undergraduate degree in Medical Management, student assistant at the Institute of General Medicine at Charité – Universitätsmedizin Berlin, with experience in qualitative research. Interviews were held in person from November 2022 to January 2023. The interviewer had no contact or relationships with participants prior to recruitment. Participants were informed that the researcher was a public health student and that the study took place within the context of a Master’s thesis. Interviewees received the interview guide prior to their participation. The locations where the interviews took place depended on the person to be interviewed and their daily work routine. The interviews were held at the participant’s workplace in the hospital or therapy centre setting without the presence of third parties.

The interviews were conducted following a semi-structured interview guide (available in English and German from the OSF project) [31]. It was designed following the four-step principle of collecting, reviewing, sorting, and subsuming interview guiding questions by Helfferich 2011 to maintain the basic principle of openness and yet provide the necessary structuring for the research interest [32]. To develop the interview guide, barriers and facilitators of prehabilitation programs from the perspective of healthcare professionals from previous studies were summarised. The interview guide covered the following aspects: organisation, cooperation and communication, time and prehabilitation implementation [24, 25, 33]. Trial-related matters that would not apply to implementation into routine care, e.g., randomisation or study documentation, were not of interest for the present study and thus not part of the interview guide because the focus lied on the prehabilitation program itself.

A pilot test of the interview guide took place with a healthcare professional involved in the PRAEP-GO trial before the first interview was conducted. Content from the (test-)interview was not included in the analysis. Interviews were expected to last from twenty minutes to a maximum of one hour. The number of interviews depended on content saturation with an expected maximum of 15 interviews. Content saturation was considered to be achieved when no new information emerged from the interviews anymore. Before recording, demographic, and occupational information was obtained to allow characterising the sample. Upon completion of each interview, the researcher prepared a postscript.

Each interview was recorded using a non-web-enabled recorder (Tschisen V90, Tschisen, China). None of the interviews were repeated. The audio recordings were transcribed by the same researcher using MAXQDA version 2022.4.0 [34]. The basis for this were the transcription rules according to Dresing and Pehl (2015) [35]. Transcripts were not sent back to any interviewee for checking or correction. Once transcribed, the audio recordings were permanently deleted. The anonymised transcripts were stored on an encrypted data folder at the research institute, to which only the conducting researcher had access.

### Data analysis

The analysis was divided into barriers and facilitators. All factors that make prehabilitation more difficult, have made it more difficult, and will make it more difficult regarding introduction into standard care were summarised in the main category “barriers”. All factors that facilitated prehabilitation or will facilitate prehabilitation regarding introduction into standard care, were summarised in the main category “facilitators”.

**Fig. 1:**
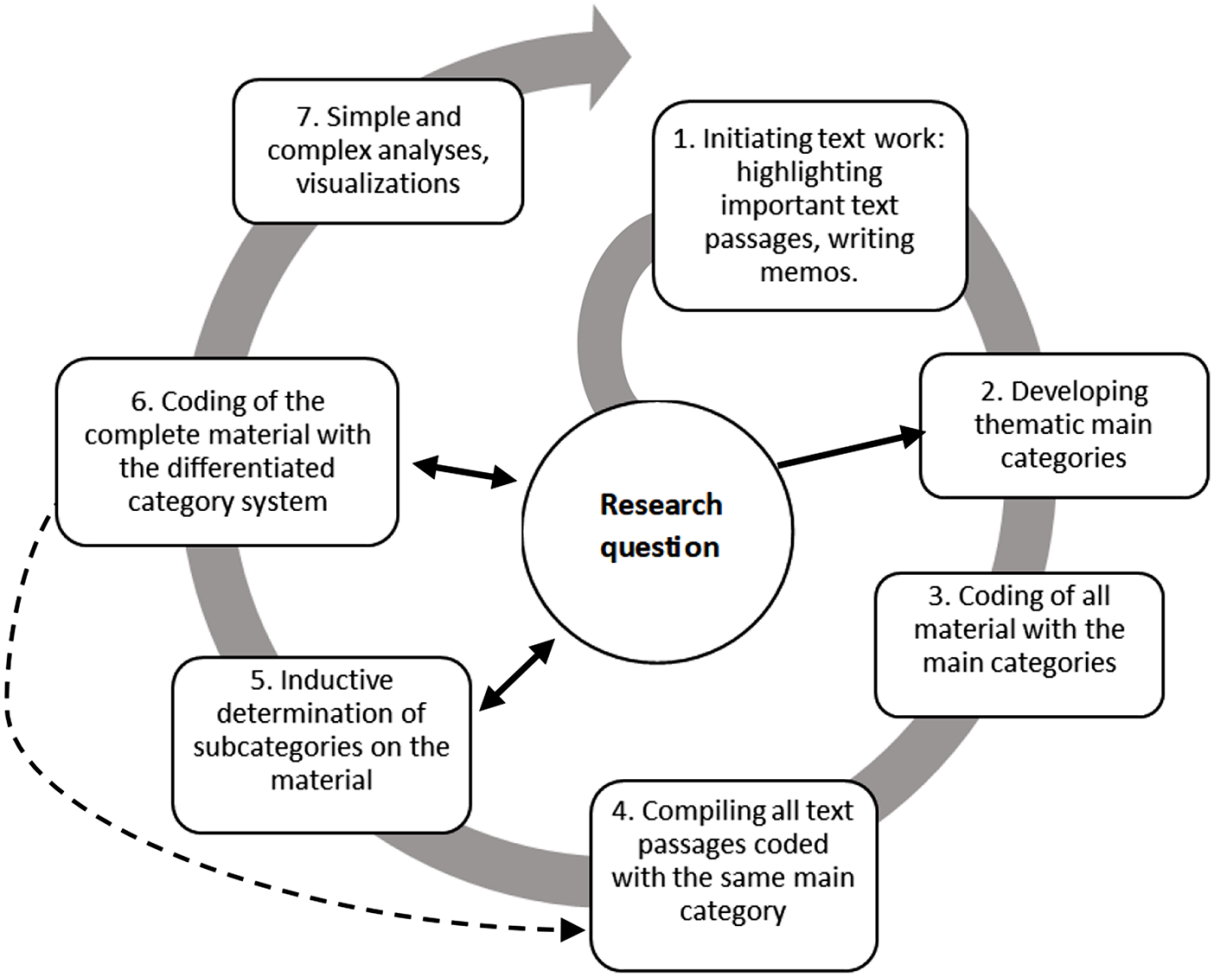
Flow model of a content structuring content analysis; own figure according to Kuckartz (2018) [36]

To categorise the interview content the software MAXQDA version 2022.4.0 was used [34]. Following transcription, the interviews were analysed using the 7-step model of content structuring content analysis according to Kuckartz (2018) (Fig. 1) [36].

The analysis approach by Kuckartz (2018) allows for both a deductive and an inductive creation of the category system. In this study, deductive categories were first created based on the results of background literature searches. The questions of the interview guide were then used as a basis to create the deductive main categories regarding barriers and facilitators (step 2): communication, time, prehabilitation and organisation. Based on these categories, the transcripts were analysed and roughly coded (step 3) and the corresponding text passages were assigned to an appropriate deductive main category using the software MAXQDA 2022 (step 4) [34]. Next, inductive subcategories based on frequently mentioned themes were added to further differentiate the deductively created main categories during the intensified text work (step 5). Finally, all transcripts were coded using the final category system (step 6) which were then synthesised and visualised (step 7). To adapt the category system to the material, the deductive main category “time” was removed, since all statements about temporal aspects could be divided into the remaining main categories.

The text passages were initially assigned by one researcher, who also conducted the interviews. Kuckartz (2018) recommends conservative coding according to Hopf and Schmidt (1993) to ensure the quality of the coding process [36, 37]. Thus, a second researcher (female, Master of Public Health student with expertise in qualitative research, qualified occupational therapist) coded the interviews independently using the pre-existing category system and a coding guide to improve the reliability of the codes (available in English and German from the OSF project) [31]. Subsequently, the coding of both researchers was compared and discussed until consensus was found. For the purpose of this publication, all participant quotations were translated verbatim into English language using DeepL software [38]. Where necessary, the grammar of supporting quotations was adapted for better comprehensibility.

### Synthesis of results

Synthesis of the final categories was guided by the updated Consolidated Framework for Implementation Research (CFIR) [39], which is a commonly used theoretical framework based on 19 published implementation science theories [40]. The updated CFIR consists of the following five main domains: (I) Innovation, (II) Outer setting, (III) Inner setting, (IV) Individuals, and (V) Implementation process [39]. These areas interact in multiple and complex ways to influence the effectiveness of an intervention’s implementation. Subordinate to the five main areas are an additional 39 underlying constructs and subconstructs, of which those most relevant to the particular setting of the PRAEP-GO trial were chosen to synthesise the final categories, and help organise and explain all outcomes of the implementation process.

## Results

### Sample

A total of 14 individuals volunteered to participate in the interviews, while the remaining 15 invitations were declined or remained unanswered. Interview requests were declined due to lack of staff and time. The demographic and occupational data of the interviewees are listed in Table 1. Interviews took between ten and 41 min (median 19 min).

**Table 1:** Baseline characteristics of interviewees

Characteristic	Participants (*N* = 14)
Gender	
Female	10
Male	4
Diverse	0
Occupational group	
Physicians	5
Therapists	7
Other	2
Years in the profession	
0–5	2
6–10	4
11–15	2
16–20	2
21–25	0
26–30	2
> 30	2
Tasks/activities in the PRAEP-GO trial	
Conducting prehabilitation	5
Management and organisation	4
Planning/consulting prehabilitation	4
Other	1
Patient contact in the PRAEP-GO trial	
Yes	14
No	0

### Barriers

A total of 215 sequences were coded under barriers. Most of the barriers experienced by health professionals related to the theme “organisation”. This subcategory included a total of 114 codes and was again subdivided into nine further subthemes or subcategories relating to organisational aspects of the prehabilitation process. The most frequently mentioned barrier was related to perceived staff shortages. One physician said on this topic:

*“That was a structural problem – at the time, there was not enough staff in orthopaedics and trauma surgery (…) for additional projects…” (B8)*.

One of the therapists mentioned:

*“(…) we weren’t well staffed on the ward, we always had staff shortages and that was critical for planning…’’ (B2)*.

Another organisational barrier was the perceived short-term planning of prehabilitation, which makes it difficult to integrate patients into the day-to-day business of prehabilitation facilities, as well as training deficits among healthcare professionals with regard to prehabilitation. Trial-related issues, such as testing, randomisation or documentation, were also frequently mentioned by the participants as a barrier, as they require additional work for healthcare staff. In addition, scheduling uncertainties in the operating room (OR) schedule and the resulting rescheduling of OR appointments were found to hamper the implementation of prehabilitation, especially for healthcare professionals from surgery. One physician commented:

*“Add to that the increased trauma surgery volume as other hospitals increasingly pull out of emergency care. This is really blowing up our elective operating rooms and patients are having to be moved like dominoes. That makes the patient unhappy, that blows up the PRAEP-GO protocol, and that’s one of the biggest hurdles we’ve had to overcome recently with this project here.” (B3)*.

The scope and effort associated with SDM conferences were also criticised, and one physician explained:

*“What really hampered it were the [SDM conferences] that are scheduled for an hour and a half and then at times when our surgery day is simply going on…” (B3)*.

The discrepancy between the current patient pathway, where patients often do not see anaesthesia at the time of initial planning of their surgery, and the ideal patient pathway, linking prehabilitation to interdisciplinary teamwork approaches within perioperative medicine, was mentioned several times as a complicating factor, e.g., by one of the physicians:

*“The concept was that the patients would be sent directly from the admission centre to anaesthesia for screening and inclusion and that didn’t work in practice. The surgeons see these patients first, make an indication and send the patients home again and anaesthetists must chase after the patients…” (B11)*.

The daily travel times for frail patients were perceived as too long and the lack of prehabilitation centres was also mentioned as a barrier.

Another subcategory with 64 sequences of “barriers” refers to the communication and cooperation of all persons involved in prehabilitation. Most frequently mentioned here was insufficient cooperation between professional groups, facilities, and medical specialties. Among other things, resistance to the concept of prehabilitation and conflicts between colleagues were reported within this subcategory. One of the physicians described this as follows:

*“There was some resistance and rejection at first because my colleagues couldn’t estimate how much work it [prehabilitation] would mean for them.” (B11)*.

Additionally, insufficient cooperation of some patients and communication deficits between patients and health professionals were mentioned as an aggravating factor.

Further barriers can be summarised within the subcategory “prehabilitation” with 37 sequences. The most frequent statement within this subcategory was that the predefined therapy plans were too rigid, resulting in patients being under or over challenged and sometimes having to idle time between therapy sessions, as described in the following quote:

*“Because some [patients] are actually fitter, they could do much more, so they do more then. They go for a walk for ages, which is not documented as a therapy session.” (B7)*.

Additionally, the time span for prehabilitation was criticised as either too long overall or too short for physiological adaptation processes. Finally, it was stated that the health status and the age of the patients made prehabilitation more difficult in some cases.

In Table 2, all identified barriers are listed by professional group.

**Table 2:** Identified barriers by professional group

Identified barriers	Physicians (*n* = 5)	Therapists (*n* = 7)	Other (*n* = 2)
**Organisation**			
Perceived personnel shortage	x	x	x
Patient pathway not adapted to incorporate prehabilitation	x		x
Scope and effort of the SDM conferences	x	x	
Lack of prehabilitation centres			x
Short term planning of prehabilitation	x	x	x
Patient transportation	x	x	x
Postponing surgery appointments	x		x
Training deficits among healthcare professionals	x	x	x
Study matters	x	x	x
**Communication & cooperation**			
Communication deficits with patients	x	x	x
Communication deficits among professionals	x	x	x
Excessive demands of patients		x	x
Limited cooperation between professionals	x	x	x
**Prehabilitation**			
Age and health status of the patients	x	x	x
Pain	x	x	x
Time period of prehabilitation	x	x	
Rigid treatment plans	x	x	x

An ‘x’ indicates that this theme was at least once mentioned by the respective professional group

### Facilitators

All factors that facilitated prehabilitation or will facilitate prehabilitation regarding introduction into regular care, were summarised in the main category “facilitators”. A total of 176 sequences were coded for this purpose. The facilitating factors are also divided into the three main themes or subcategories of communication & cooperation, prehabilitation, and organisation.

A total of 77 sequences referred to the subcategory “organisation”. The most frequently mentioned facilitating factor was the need for optimising the patient pathway, including the simplification and standardisation of the prehabilitation process. One physician said in this regard:

*“I believe that the overall process should be simplified, both in terms of preparing people and asking who is a candidate for prehabilitation, as well as the measures that are taken.” (B10)*.

Health professionals stated that by introducing prehabilitation, many complicating factors of prehabilitation, such as rigid therapy plans to ensure comparability of data, would automatically be eliminated, making prehabilitation easier. One of the physicians argued:

*“I think if this is incorporated into standard care, then patients are treated more pragmatically and not so according to a standard, according to a protocol. I believe that if a patient has the motivation to stay longer, to do self-exercises, then no one will forbid this patient to do so.” (B11)*.

Another frequently mentioned facilitating factor regarding the organisation of prehabilitation was more personnel resources. The SDM conferences were also identified to play an important role in prehabilitation, as they facilitate communication, information sharing, and goal setting among health professionals and with patients. Participants called for more time to plan prehabilitation and involve prehabilitation facilities and suggested the increased use of smart IT solutions and technology within the prehabilitation process to facilitate certain procedures. There was also a call for a clearer documentation within the prehabilitation process.

With a total of 60 coded sequences in the subcategory “prehabilitation” most of the statements here referred to a more individualised therapy design. One of the therapists suggested in this regard:

*“It would probably make more sense if you had a little more time or if you could get away from this rigid regulation of breaks, for example in strength training (…)” (B5)*.

The choice of outpatient, inpatient, day-care or home visit prehabilitation was identified a facilitating factor. Some statements referred to the fact that a longer prehabilitation period and thus fewer therapy units per week would have a beneficial effect on the prehabilitation and physiological adaptation process. The form of multimodal prehabilitation was also addressed as crucial. One physician praised the concept as follows:

*“… The fact that this prehabilitation [programme] is multimodal is really undisputed. We have a very, very wide range and I am glad and proud that we can offer this here and I think the patients are also very taken with the possibility of really being checked through and getting support, which is also worthwhile.” (B11)*.

Well-trained therapists and a full hour of therapy time for each patient would ensure that prehabilitation measures can be carried out optimally and quality standards can be better maintained.

Prehabilitation would be further facilitated by stronger evidence, so that the concept becomes more established and known, both by health professionals and patients. A physician commented:

*“If the evidence [base] improves and we can show how the patients benefit from it [prehabilitation] and the concept is better or more accepted in people’s minds (…), I believe that they [the patients] will participate. Because now we must convince people of the concept [of prehabilitation] who didn’t even know about it before and already have an idea of how rehabilitation [after the surgery] works. But if they already know that there are also prehabilitation programmes, (…) then they [the patients] might come to us with [an idea of] the concept and then their willingness to participate will be certainly greater…” (B8)*.

A total of 39 codes were assigned to the subcategory “communication and cooperation”. Here, close cooperation between different professional groups and institutions was most frequently mentioned as a facilitating factor. One of the physicians shared this view:

*“I think it’s conducive and important to really have all the teams on board. That was relatively clear in our case, that if you then work together with senior physicians who are also on board, it works well.” (B8)*.

Effective and efficient communication among each other was also found to have a facilitating effect on prehabilitation. There was a desire for fixed contact persons and a fixed team that is responsible for the implementation of prehabilitation.

In Table 3, all identified facilitators are listed by professional group.

**Table 3:** Identified facilitators by professional group

Identified facilitators	Physicians (*n* = 5)	Therapists (*n* = 7)	Other (*n* = 2)
**Organisation**			
Sufficient personnel resources	x	x	x
Optimisation of patient pathway	x	x	x
Standardised structures and organisation of prehabilitation	x	x	x
Use of IT and technology	x		x
More planning time for prehabilitation	x	x	x
Compressed documentation	x	x	
Concept of the SDM Conferences	x	x	x
Implementation of prehabilitation in regular care	x		x
**Communication and cooperation**			
Good communication between professionals	x	x	x
Fixed contact person/permanent team	x	x	x
Close cooperation between different professional groups	x	x	x
**Prehabilitation**			
Positive impact of prehabilitation on patients	x	x	x
Committed patients		x	
Well trained therapists		x	
Longer prehabilitation period + fewer units per week	x	x	
Individual therapy design	x	x	
Multimodal prehabilitation	x	x	x
One hour therapy time		x	
Choice of setting (outpatient/inpatient/partial inpatient/home visits)	x	x	x
Manual for therapists		x	
Proof of evidence	x		

An ‘x’ indicates that this theme was at least once mentioned by the respective professional group

### Synthesis of results

**Fig. 2:**
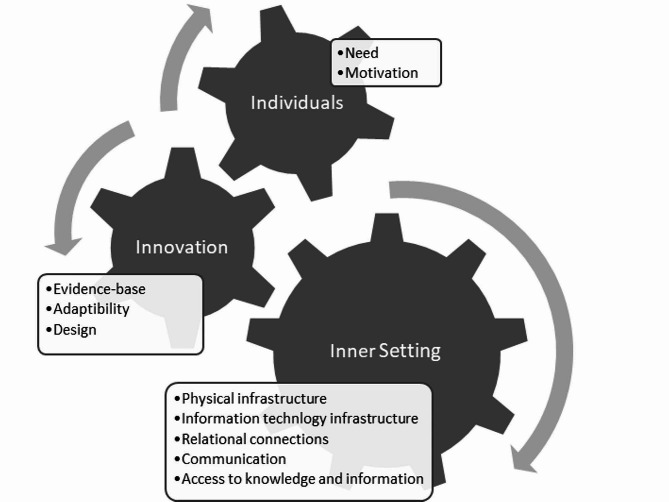
Synthesis of results using the updated CFIR framework; own figure

The areas most relevant for implementation of the PRAEP-GO intervention were summarised by placing the results of the present work within three of the five domains of the updated CFIR framework (Innovation, Inner Setting, and Individuals; Fig. 2) [39].The full results can be found in appendix B.

At the innovation level, the focus should be on the evidence base, adaptability, and prehabilitation programme design. We identified a need for good communication and high-quality information exchange within the inner setting and across its boundaries, as well as access to knowledge and information for innovation deliverers.

At the level of the inner setting, meaning hospitals or prehabilitation facilities in which the PRAEP-GO intervention takes place, necessary infrastructure must be in place to support the functional performance of the inner setting and enable the implementation of the innovation. This includes physical infrastructure, such as the presence of sufficient prehabilitation centres, information technology infrastructure to support electronic documentation, data storage and management, and the work infrastructure. The work infrastructure refers to the organisation of tasks and responsibilities, but also to the availability of sufficient human resources. Relational connections are another prerequisite for the successful implementation of prehabilitation in the internal setting. This refers to high-quality formal and informal relationships, networks, and teams, also beyond the boundaries of the inner setting, such as good cooperation among professionals or with patients.

At the CFIR level of the individual’s domain, the focus is on the roles and characteristics of the individuals involved in prehabilitation. In this domain, the PRAEP-GO intervention has to meet the needs of the patients and the motivation or commitment of the individuals is a decisive factor.

## Discussion

A total of 14 interviews were conducted with seven therapists, five physicians, and two employees from other professional groups. All identified barriers and facilitating factors could be assigned to the themes of organisation, prehabilitation, cooperation and communication between healthcare professionals and with patients. Almost all the barriers and facilitating factors mentioned were addressed by several or even all professional groups and are therefore relevant for them. The consensus among different professional groups, for example about barriers, underscores their priority and relevance across professions. However, there were some aspects that reflect the specific need of the occupational group. For example, facilitating factors like “proof of evidence” were only addressed by physicians and “manual for therapists” only by therapists.

Most of the space within the interviews, both for barriers and facilitating factors, was taken up by statements about purely organisational aspects of prehabilitation. It can be concluded that organisational structures for the implementation of innovative programmes, such as the PRAEP-GO intervention, are the basis for the smooth running of such projects. Adaptations in the patient pathway are therefore a prerequisite [41], and should incorporate prehabilitation into the existing interdisciplinary teamwork approaches within perioperative medicine. In 2022, van der Zanden et al. also noted that organisational aspects, such as sufficient human resources, especially for good coordination of the program, must be in place as a prerequisite for the successful implementation of a prehabilitation program, so that physicians and therapists have sufficient capacity for the actual prehabilitation [24]. This underlines the need for an optimal allocation of resources and a reduction of excessive bureaucracy to free up capacities in the work force and reduce perceived staff shortages. According to Arora et al. (2018) the contextual readiness of organisations in terms of leadership support, flexibility of existing surgical practice culture, data processing capabilities, and generally having sufficient resources for successful implementation of prehabilitation programmes is critical to their success [42].

Regarding prehabilitation and its implementation, an individually designed and multimodal prehabilitation is essential. According to Beck et al. (2021), recommendations that are too general are an obstacle to patient adherence if they are perceived as irrelevant or unimportant [43]. In addition, the training programmes must correspond to the individual needs and abilities of the patients [24, 25], e.g. the number of sessions per week that are tolerable for the individual patient. From the interviews of the present work, an individual therapy design does not only concern the needs of the patients, but therapists also wish for a more individual therapy design to be able to optimally prepare their patients for their operation with their own methods. In addition, a more flexible therapy design – in comparison to the rigid demands of a standardised intervention within a clinical trial – can avoid over- and under-challenging of patients as well as idle time within and between therapy sessions.

Another aspect that concerns the implementation of prehabilitation relates to its time span. If the patient has an indication that allows the period before surgery to be extended, prehabilitation could also be extended so that physiological adaptation processes can occur more intensively and sustainably until surgery. Extending the prehabilitation period if possible is also recommended by Beck et al. in their study from 2018 on prehabilitation in cancer care [43]. Evidence, or the perception of evidence by participating health professionals, is also the key factor for successful team engagement [42]. Adequately demonstrating the effectiveness of prehabilitation could also make it easier to acquire financial support and create a greater willingness to implement prehabilitation among health professionals and within your facilities [44].

A lack of commitment on the part of health professionals also affects their cooperation and communication with each other and with patients. For example, if the treating surgeons do not fully agree with the concept of prehabilitation, this can lead to misunderstandings, misaligned goals within the treatment team, and lack of patient engagement, according to Ng et al. (2022) [45]. For the implementation of complex interventions such as PRAEP-GO, it is important that different professional groups across different areas work together as a team. Especially for patient safety the performance of the team is crucial [46]. Skill mix can only take place with good inter- and intra-professional cooperation of the team members [47].

Physicians in particular have an important leadership role and influence on the motivation of their patients [48]. This is critical for the diffusion, dissemination, and implementation of prehabilitation at the micro (clinical integration) and meso (professional and organisational integration) levels, as well as at the macro level (system integration) [41, 49]. The prerequisite for cooperation both between health professionals and between patients is sufficient and goal-oriented communication. In the study by van der Zanden et al. (2022), communication deficits, which are usually accompanied by information deficits, also represent a barrier in the context of prehabilitation [24].

### Limitations

Despite following rigorous methodology including the prospective registration of this interview study, some limitations at the study level apply that need to be considered when interpretating the results. First, a saturation of content could not fully be achieved as the recruitment of interview partners had to be discontinued after the 14th interview due to limited resources for the interviews. For the same reason, the recruitment of interview participants took place exclusively in the Berlin and Brandenburg area, although the project also takes place in study centres in other parts of Germany whose perspective could not be captured. Another limitation is that the professional group of therapists predominates in the sample and may have influenced the focus of the barriers and facilitators mentioned. The professional group of nutritionists was also approached during the recruitment process but did not participate, so their perspective could not be represented.

Furthermore, dependability might have been affected by the fact that the interviews and developing the category system was performed by one researcher. Although the interviews were coded independently and the coding was discussed in a team, the researcher who created the coding system and conducted and transcribed the interviews might have had more influence on the final coding than the other coder. In addition, the theoretical framework used was selected after the interviews had been conducted and coded. Lastly, the generalisability of the findings to other populations and contexts should be viewed cautiously.

## Conclusions

The aim of the study was to identify barriers and facilitators to the implementation of a prehabilitation programme for frail people aged 70 years and older in Germany from the perspective of the health professionals involved. The findings were synthesised using the theoretical CFIR framework, which highlighted the implications of the study for the areas of the innovation, internal setting, and individuals. Identified barriers were communication deficits and insufficient cooperation between healthcare professionals and with patients. Age, health status of the patients, pain and overly rigid treatment plans were also barriers, as well as lack of staff, the patient pathway not being adapted to incorporate prehabilitation, the scope and effort of SDM conferences, lack of prehabilitation centres, short-term planning, transport of patients, postponement of surgery, training deficits and time-consuming study matters, such as testing and documentation. Facilitators for a successful implementation of prehabilitation programmes, such as the PRAEP-GO programme, are sufficient organisational infrastructure, human resources, and access to knowledge and information for innovation providers. The development of an adaptable and individualised treatment design that must meet patients’ individual needs and abilities is critical. Good cooperation, communication, and quality information exchange among professionals and with patients are also critical. To further convince professionals and patients of the concept of prehabilitation, more research needs to be conducted on this topic to build a solid evidence base. This will ensure greater awareness and thus more motivation and cooperation among professionals and patients.

### Electronic supplementary material

Below is the link to the electronic supplementary material.

Supplementary Material 1

## Data Availability

Due to the data protection regulations of the study, no raw data (i.e. transcripts) can be made available. However, a complete coding guide including quotations from the interviews is available from the OSF project (doi: 10.17605/OSF.IO/Q59P8).

## Abbreviations

CFIR: Consolidated Framework for Implementation Research
COREQ: Consolidated criteria for Reporting Qualitative research
MAXQDA: Software package for qualitative data analysis
PRAEP-GO: Prehabilitation of elderly frail or pre-frail patients prior to elective surgery ─ a randomised controlled multicenter study
SDM conference: Shared decision-making conference
