# Supplementary material for: Barriers and facilitators to the implementation of prehabilitation for elderly frail patients prior to elective surgery: a qualitative study with healthcare professionals
Source: BMC Health Serv Res. 2024 Apr 26;24:536. doi: 10.1186/s12913-024-10993-2 (PMC11046874; doi:10.1186/s12913-024-10993-2)
Supplement: Supplementary file 1 — Supplementary Material 1 [file 12913_2024_10993_MOESM1_ESM.docx]

# Supplementary material

## Appendix A: Interview guide

**Research question**

What barriers and facilitating factors did experts from different health care professions involved in the PRAEP-GO project experience in implementing prehabilitation as a new form of care?

**Flowchart**

**1. Before the interview:**

- Review of the signed consent and privacy statement
- Conversation starter:
  - Introduction as a master student of the BSPH
  - Framework data for the interview (data protection, duration, etc.)
  - Note that there are no wrong answers and detailed answers to the topics are desired
  - (Subjective) own experience, attitudes and experiences of interest
  - Clarification of open questions

**Possible wording:**

*"Good Mr./Mrs. (title) .... nice to see you.*

*I would like to welcome you to our interview about barriers and facilitating factors in the prehabilitation process of the PRAEP-GO project and thank you for your willingness to participate in the study. My name is Tamina Fuchs, I am a master student at the Charité Berlin and I am studying Public Health in the 4th semester.*

*In this survey I am committed to confidentiality and no personal data from you will be included in the subsequent work. I am only interested in your expert knowledge regarding prehabilitation and the PRAEP-GO project. Participation in the interview is voluntary and you may withdraw your consent at any time. There are no wrong answers in this interview and detailed narratives are encouraged. I estimate the duration of the interview to be approximately half an hour to one hour at the most. Depending on how much time you have available.*

*Do you have any other questions about the project?*

*If they agree with everything, I would ask you a few demographic and job-related questions at the beginning. After that, the actual interview will begin and only then will the recording of the interview start.*

**2. Demographic and occupational questions (before audio recording)**

**3. Interview conversation**

- Dictation machine - pronounce information: "The dictation machine is now on!"
- Checking whether dictation machine is really on

**4. End of conversation**

- Dictation machine - pronounce information: "The dictation machine is now off!"
- Brief reflection - how did you feel about the conversation?
- Thanks and farewell

**5. After the interview:** *fill in the postscript*

**Interview conversation**

**Demographic and occupational issues**

1. To which gender do you assign yourself?
2. What are your tasks/activities within the PRÄP-GO project?
3. Which professional group do you belong to?
4. How long have you been working in your profession?

**Interview Questions:**

Barriers

*Organization*

a) From your perspective, what was the biggest barrier that made the prehabilitation process difficult?
b) What other complicating factors were there?

Topics taken up from literature

c) How did you feel about staffing in your area prehabilitation process?
d) How would you rate planning and organization in your area prehabilitation process?

e) How did you feel about the communication between the different areas/centers involved in the prehabilitation process?
f) What factors made communication between stakeholders difficult or easier?
g) (How did you feel about the communication with the patients*).
*Only ask experts with patient contact*

*Communication*

h) In general, how do you estimate the amount of time available for prehabilitation?
i) How would you rate the time you have/had in your role, in the prehabilitation process?

*Time*

j) (Which types of prehabilitation interventions do you consider most appropriate and which more inappropriate?) *Only ask treatment providers*
k) (In your opinion, which location is suitable for conducting prehabilitation measures and which is not?) *Only ask treatment providers*

*Implementation*

Facilitating factors

l) What were conducive factors that most facilitated the prehabilitation process?
m) What would further facilitate prehabilitation and its uptake into mainstream care?

Conclusion

n) If you could change one thing about the prehabilitation process, what would it be?
o) Is there anything else we haven't talked about yet that is important to you?

**Postscript**  pseudonym/participant*: _______________

Did the interview take place on time?

___________________________________________________________________

How was the video/sound/call/recording quality?

___________________________________________________________________

Were there any disruptions/interruptions?

___________________________________________________________________

What mood was perceived/ How does the interviewee appear to me?

___________________________________________________________________

___________________________________________________________________

What atmosphere has built up?

___________________________________________________________________

___________________________________________________________________

How did I feel as an interviewer? Were there any special moments/ feelings/ ...?

___________________________________________________________________

___________________________________________________________________

Were there any other special features?

___________________________________________________________________

## Appendix B: COREQ Checklist

| **Topic** | **Item No.** | **Guide Questions/Description** | **Reported on**  **Page No.** |
| --- | --- | --- | --- |
| **Domain 1: Research team**  **and reﬂexivity** | | | |
| *Personal characteristics* | | | |
| Interviewer/facilitator | 1 | Which author/s conducted the interview or focus group? | 7 |
| Credentials | 2 | What were the researcher’s credentials? E.g. PhD, MD | 7 |
| Occupation | 3 | What was their occupation at the time of the study? | 7 |
| Gender | 4 | Was the researcher male or female? | 7 |
| Experience and training | 5 | What experience or training did the researcher have? | 7 |
| Relationship with  participants | | | |
| Relationship established | 6 | Was a relationship established prior to study commencement? | 7 |
| Participant knowledge of the interviewer | 7 | What did the participants know about the researcher? e.g. personal goals, reasons for doing the research | 7 |
| Interviewer characteristics | 8 | What characteristics were reported about the inter viewer/facilitator? e.g. Bias, assumptions, reasons and interests in the research topic | 7 |
| **Domain 2: Study design** | | | |
| *Theoretical framework* | | | |
| Methodological orientation and Theory | 9 | What methodological orientation was stated to underpin the study? e.g. grounded theory, discourse analysis, ethnography, phenomenology, | 8 |
| Participant selection | | | |
| Sampling | 10 | How were participants selected? e.g. purposive, convenience,  consecutive, snowball | 6 |
| Method of approach | 11 | How were participants approached? e.g. face-to-face, telephone, mail,  email | 6 |
| Sample size | 12 | How many participants were in the study? | 10 |
| Non-participation | 13 | How many people refused to participate or dropped out? Reasons? | 10 |
| *Setting* | | | |
| Setting of data collection | 14 | Where was the data collected? e.g. home, clinic, workplace | 7 |
| Presence of non -participants | 15 | Was anyone else present besides the participants and researchers? | 7 |
| Description of sample | 16 | What are the important characteristics of the sample? e.g. demographic data, date | 11 |
| *Data collection* | | | |
| Interview guide | 17 | Were questions, prompts, guides provided by the authors? Was it pilot tested? | 7 |
| Repeat interviews | 18 | Were repeat inter views carried out? If yes, how many? | 8 |
| Audio/visual recording | 19 | Did the research use audio or visual recording to collect the data? | 8 |
| Field notes | 20 | Were ﬁeld notes made during and/or after the interview or focus group? | 7 |
| Duration | 21 | What was the duration of the interviews or focus group? | 10 |
| Data saturation | 22 | Was data saturation discussed? | 7, 22 |
| Transcripts returned | 23 | Were transcripts returned to participants for comment and/or correction? | 8 |
| **Domain 3: analysis and findings** | | | |
| Data analysis | | | |
| Number of data coders | 24 | How many data coders coded the data? | 9 |
| Description of the coding tree | 25 | Did authors provide a description of the coding tree? | 9 |
| Derivation of themes | 26 | Were themes identified in advance or derived from the data? | 9 |
| Software | 27 | What software, if applicable, was used to manage the data? | 9 |
| Participant checking | 28 | Did participants provide feedback on the findings? | 8 |
| Reporting | | | |
| Quotations presented | 29 | Were participant quotations presented to illustrate the themes/findings? Was each quotation identified? e.g. participant number | 11-16 |
| Data and ﬁndings consistent | 30 | Was there consistency between the data presented and the findings? | 10-20 |
| Clarity of major themes | 31 | Were major themes clearly presented in the findings? | 19-20 |
| Clarity of minor themes | 32 | Is there a description of diverse cases or discussion of minor themes? | 19-20 |

## Appendix C: Synthesis of results using the updated CFIR framework

| **CFIR Domain** | **Barriers** | **Facilitators** |
| --- | --- | --- |
| ***Innovation*** |  |  |
| Evidence-Base | n.a. | Proof of better evidence |
| Adaptability | n.a. | Individual therapy design |
|  |  | Multimodal prehabilitation |
|  |  | Choice of outpatient/inpatient/partial inpatient/home visits |
| Design | Time period of prehabilitation | One hour therapy time |
|  | Rigid Treatment plans | Longer prehabilitation period + fewer units |
|  | Linking prehabilitation to the department of anaesthesiology | Manual for therapists |
| ***Inner setting*** |  |  |
| Physical infrastructure | Lack of prehabilitation centres | n.a. |
|  | Patient transportation |  |
| Information technology infrastructure | n.a. | Use of IT and technology |
| Work infrastructure | Personnel shortage | More personnel resources |
|  | Postponing surgery appointments | Compressed documentation |
|  | Scope and effort of the SDM conferences | Concept of the SDM conferences |
|  | Study matters | Implementation of prehabilitation into regular care |
|  | Short term prehabilitation planning | More planning time for prehabilitation |
|  |  | Process optimizations |
|  |  | Fixed structures and standardised organisation |
| Relational connections | Insufficient cooperation between professionals | Close cooperation between different professional groups |
|  |  | Fixed contact person/permanent team |
|  |  |  |
| Communication | Communication deficits among professionals | Better communication between professionals |
|  | Communication deficits with patients |  |
| Access to knowledge and information | Training decifits among professionals | Well trained therapists |
| ***Individual*** |  |  |
| Need | Age and health status of the patients | n.a. |
|  | Pain | n.a. |
